# Supplementary material for: An Insight into the Role of Reactant Structure Effect in Pd/C Catalysed Aldehyde Hydrogenation
Source: Nanomaterials (Basel). 2022 Mar 9;12(6):908. doi: 10.3390/nano12060908 (PMC8955477; doi:10.3390/nano12060908)
Supplement: Supplementary file 1 [file nanomaterials-12-00908-s001.zip › nanomaterials-1613244-supplementary.pdf]

Supplementary Materials

An Insight into the Role of Reactant Structure Effect in Pd/C Catalysed Aldehyde Hydrogenation

Marta Stucchi [1], Francesca Vasile [1], Stefano Cattaneo [1], Alberto Villa [1], Alessandro Chieregato [2], Bart D. Vandegehuchte [3] and Laura Prati [1,*]

[1] Chemistry Department, University of Milan, Via Golgi 19, 20133 Milan, Italy; marta.stucchi@unimi.it (M.S.); francesca.vasile@unimi.it (F.V.); stefano.cattaneo2@unimi.it (S.C.); alberto.villa@unimi.it (A.V.)
[2] TotalEnergies Research Center—Qatar (TRCQ), Qatar Science & Technology Park, Al Gharrafa, Doha P.O. Box 9803, Qatar; alessandro.chieregato@totalenergies.com
[3] TotalEnergies One Tech Belgium, Zone Industrielle Feluy C, B-7181 Seneffe, Belgium; bart.vandegehuchte@totalenergies.com
* Correspondence: laura.prati@unimi.it; Tel.: +39-02503-14357

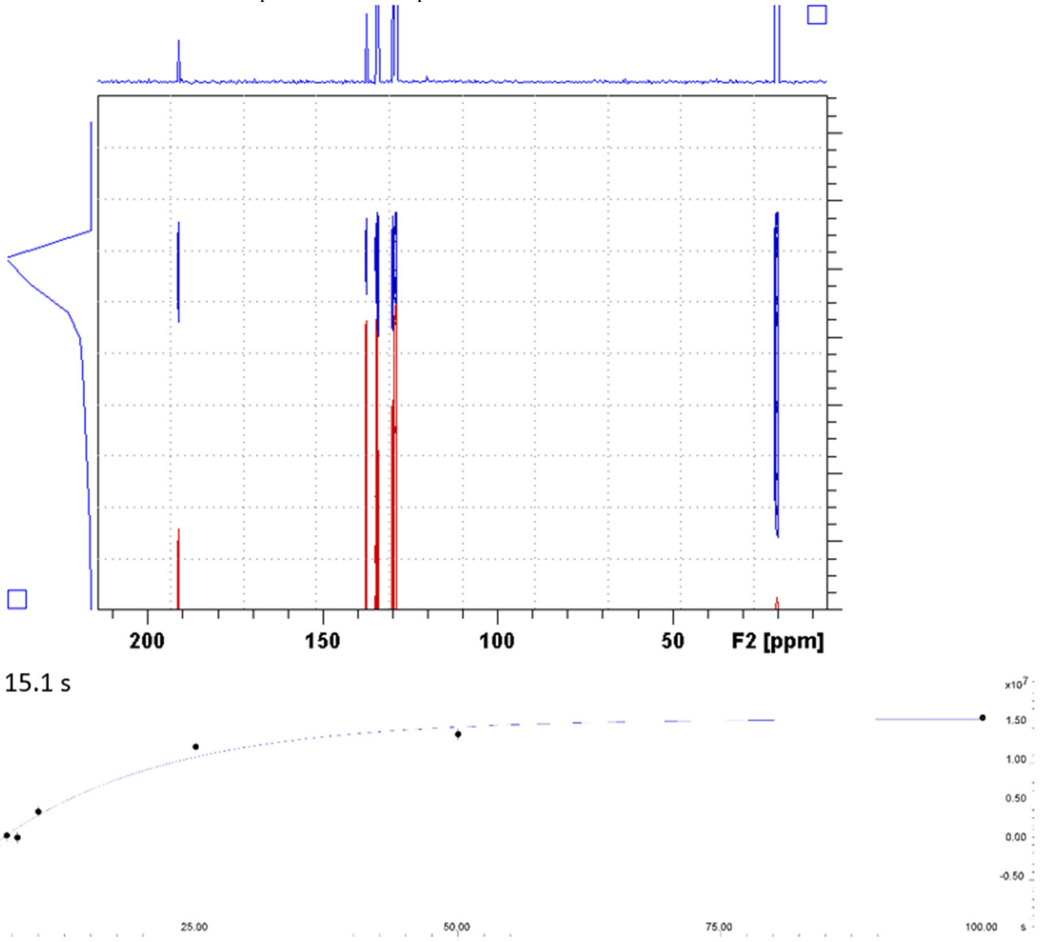

Figure S1. Analysis of Benzaldehyde in p-xylene (T1bulk). Pseudo-2D NMR experiment for T1 measurement using inversion recovery sequence (**top**) and analysis of the inversion recovery curve for the aldehydic carbon (**bottom**).

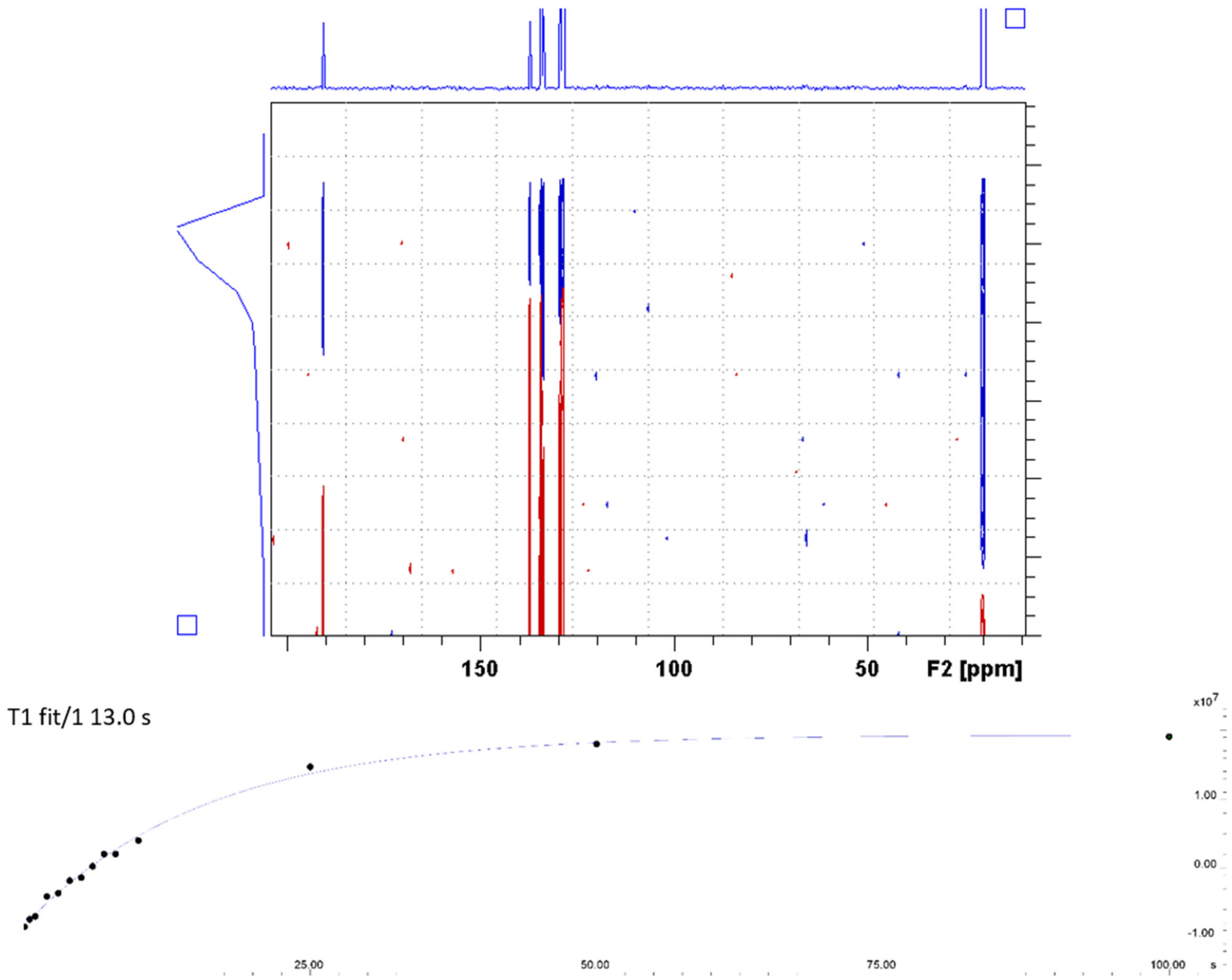

Figure S2. Analysis of Benzaldehyde in p-xylene in presence of GNP. Pseudo-2D NMR experiment for T1 measurement using inversion recovery sequence (**top**) and analysis of the inversion recovery curve for the aldehydic carbon (**bottom**).

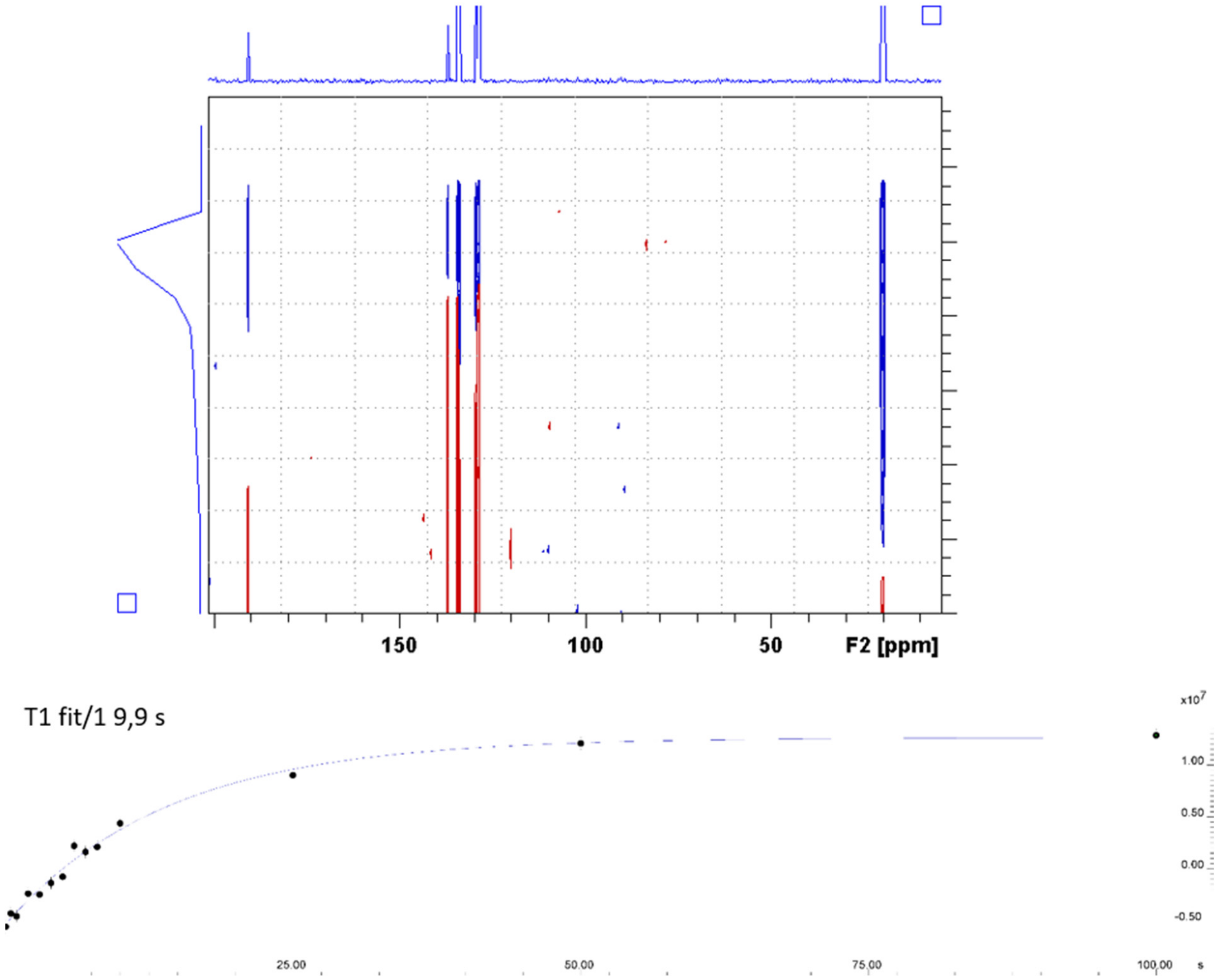

Figure S3. Analysis of Benzaldehyde in p-xylene in presence of Pd-GNP. Pseudo-2D NMR experiment for T1 measurement using inversion recovery sequence (**top**) and analysis of the inversion recovery curve for the aldehydic carbon (**bottom**).

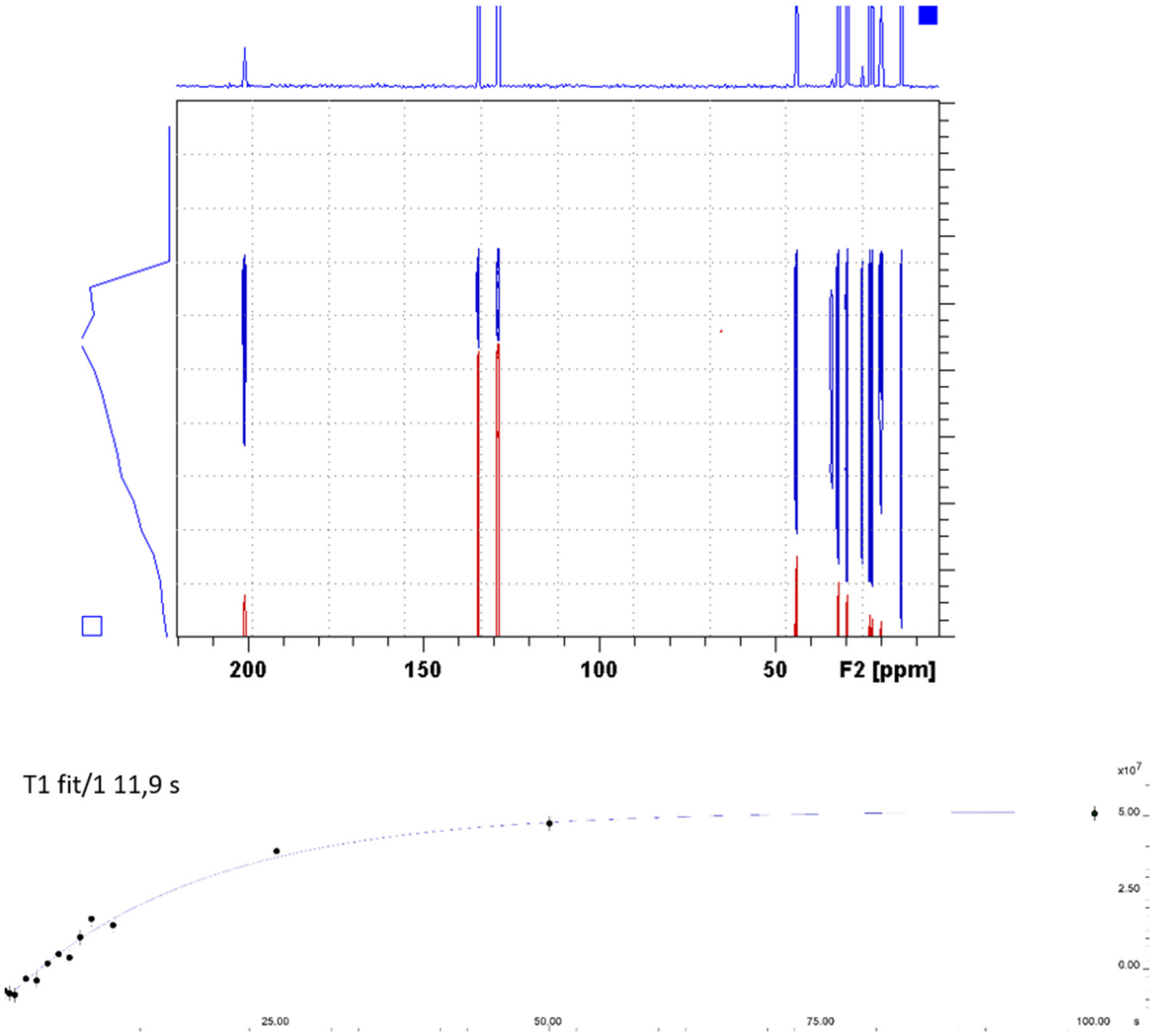

Figure S4. Analysis of Octanal in p-xylene (T1bulk). Pseudo-2D NMR experiment for T1 measurement using inversion recovery sequence (**top**) and analysis of the inversion recovery curve for the aldehydic carbon (**bottom**).

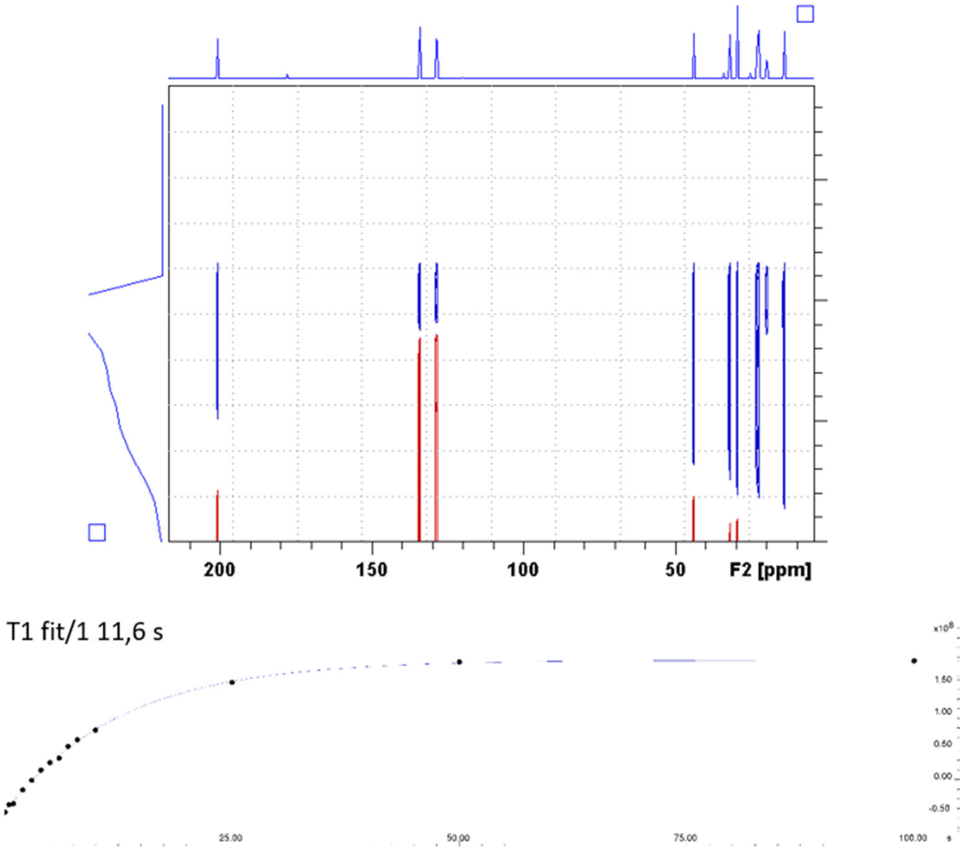

Figure S5. Analysis of Octanal in p-xylene in presence of GNP. Pseudo-2D NMR experiment for T1 measurement using inversion recovery sequence (**top**) and analysis of the inversion recovery curve for the aldehydic carbon (**bottom**).

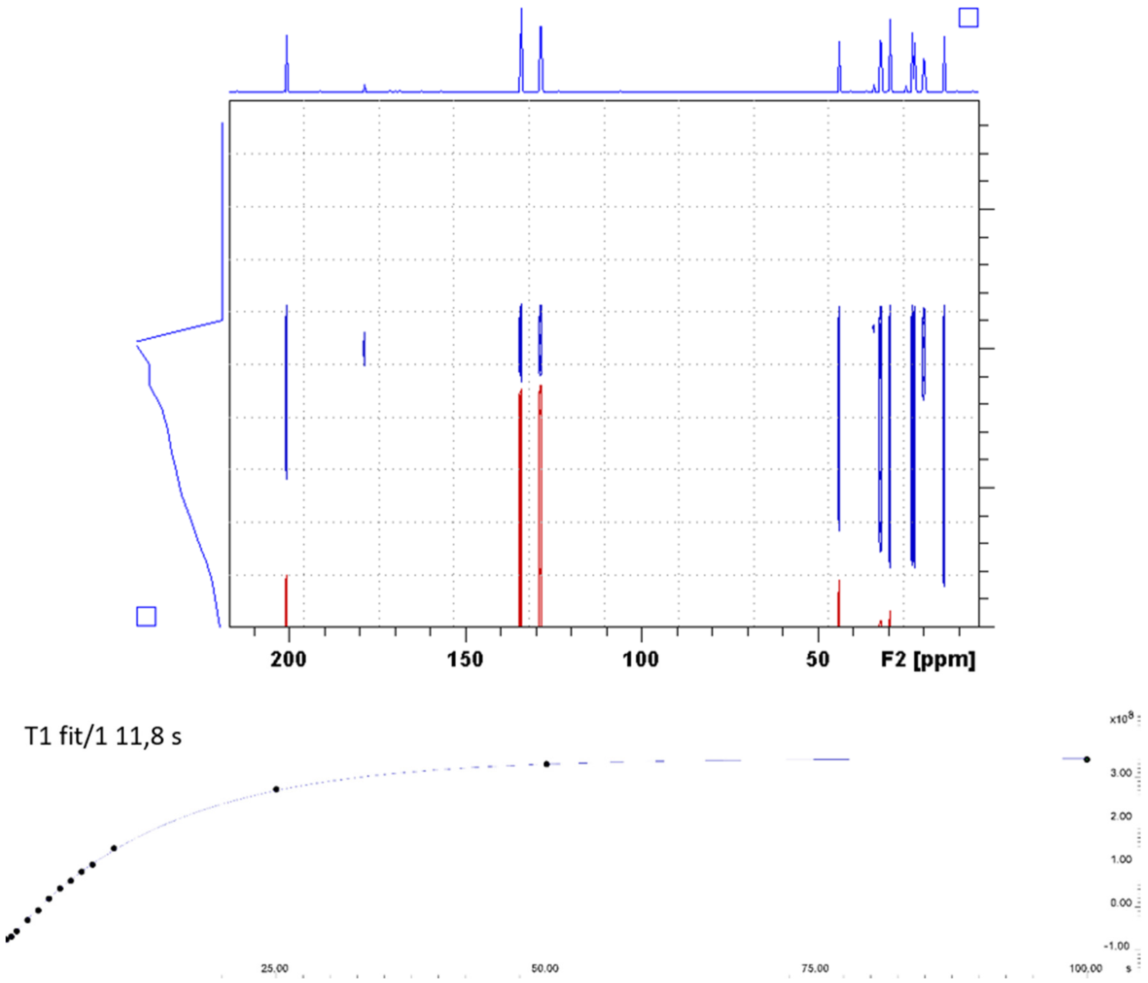

Figure S6. Analysis of Octanal in p-xylene in presence of Pd-GNP. Pseudo-2D NMR experiment for T1 measurement using inversion recovery sequence (**top**) and analysis of the inversion recovery curve for the aldehydic carbon (**bottom**).

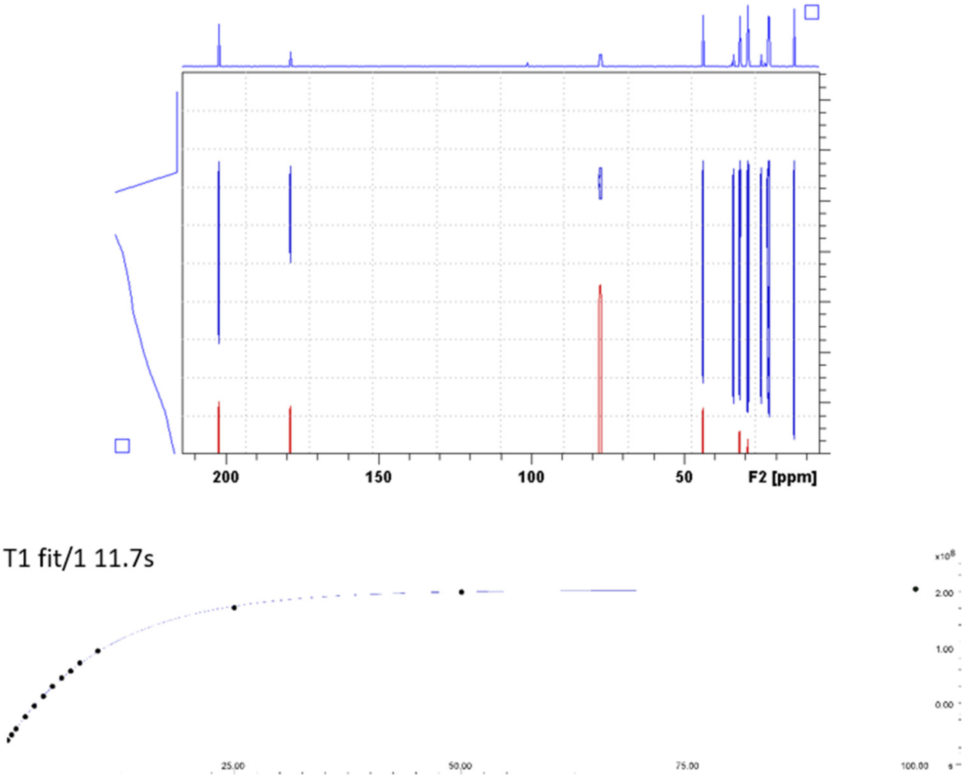

Figure S7. Analysis of Octanal in $CDCl_3$ (T1bulk). Pseudo-2D NMR experiment for T1 measurement using inversion recovery sequence (**top**) and analysis of the inversion recovery curve for the aldehydic carbon (**bottom**).

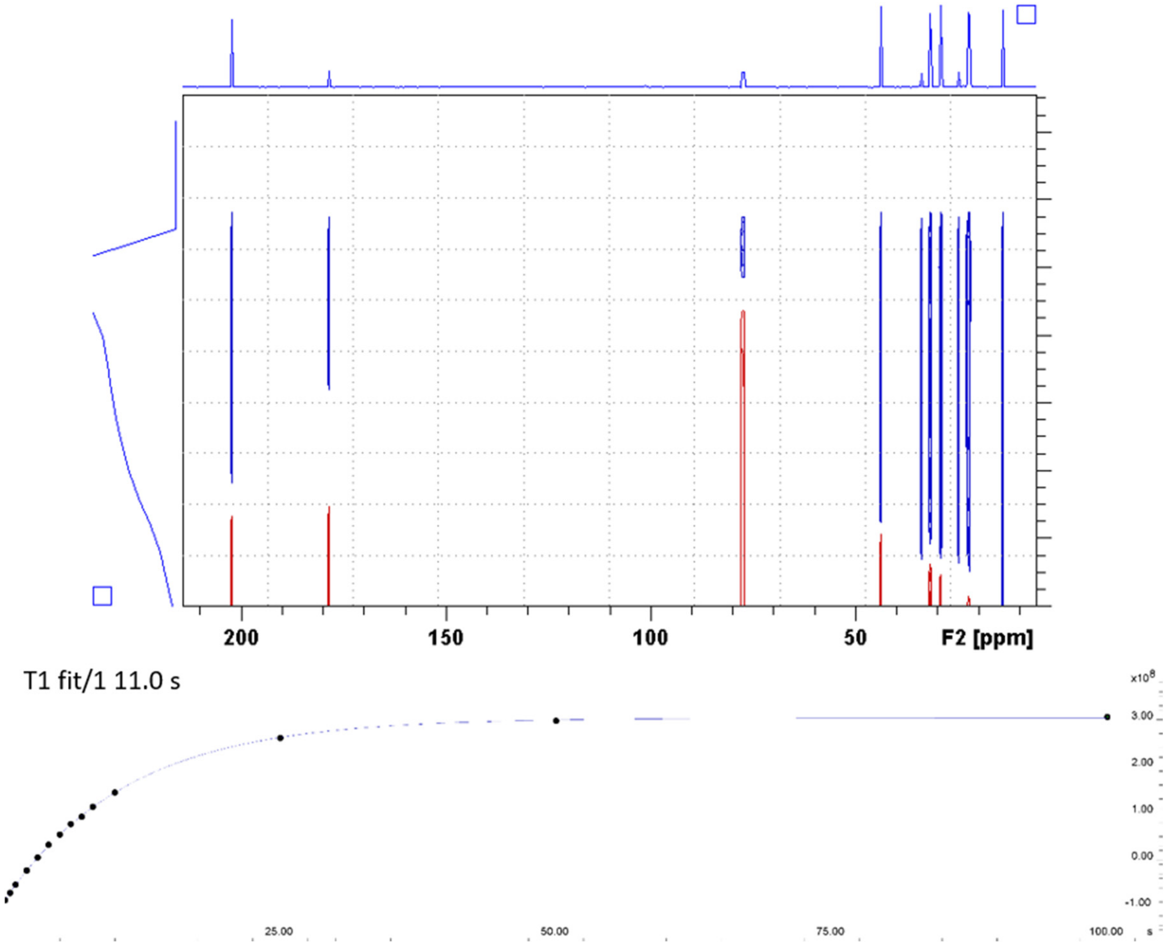

Figure S8. Analysis of Octanal in $CDCl_3$ in presence of GNP. Pseudo-2D NMR experiment for T1 measurement using inversion recovery sequence (**top**) and analysis of the inversion recovery curve for the aldehydic carbon (**bottom**).

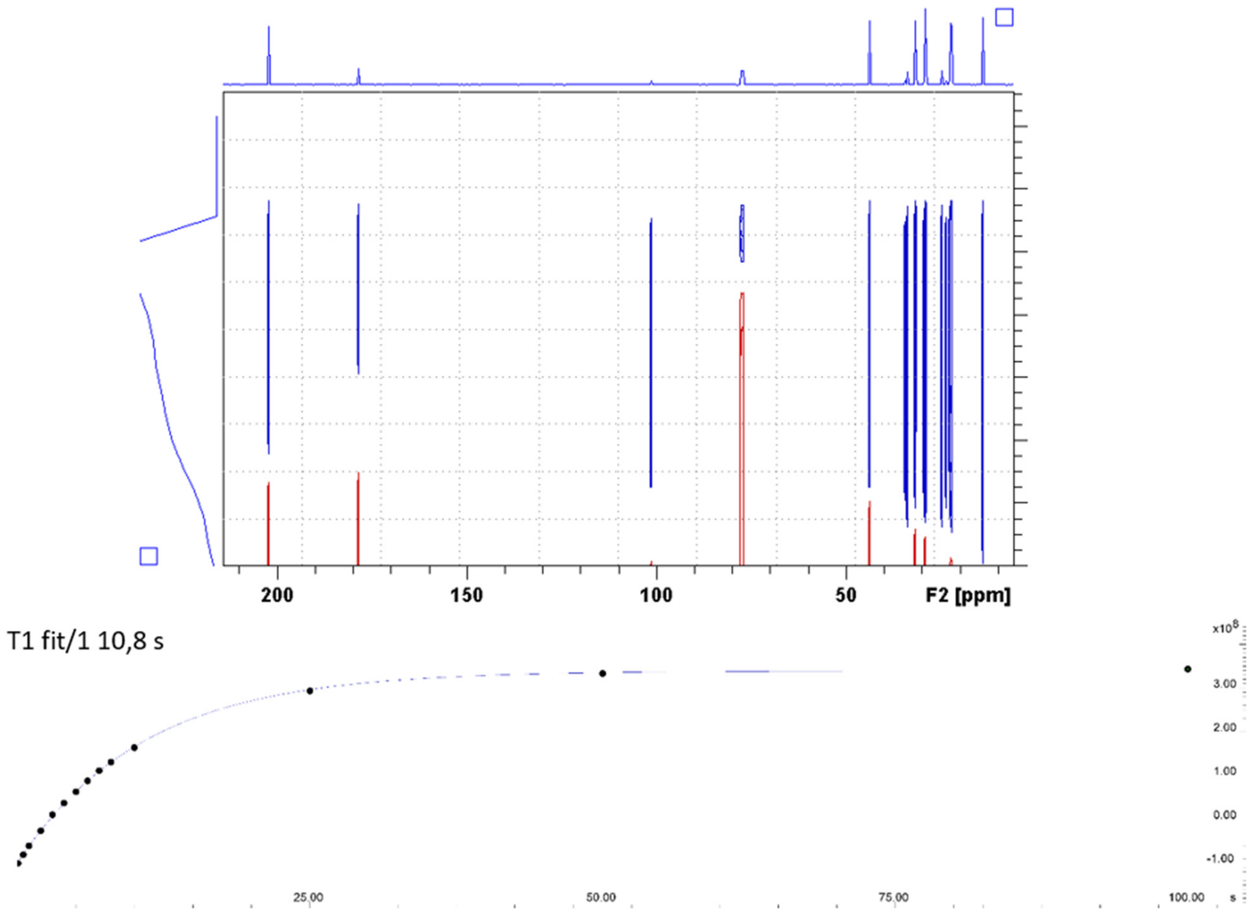

Figure S9. Analysis of Octanal in $CDCl_3$ in presence of Pd-GNP. Pseudo-2D NMR experiment for T1 measurement using inversion recovery sequence (**top**) and analysis of the inversion recovery curve for the aldehydic carbon (**bottom**).
